# Current and Future Applications of the Kambin’s Triangle in Lumbar Spine Surgery

**DOI:** 10.7759/cureus.25686

**Published:** 2022-06-06

**Authors:** Romaric Waguia, Nithin Gupta, Katherine L Gamel, Alvan Ukachukwu

**Affiliations:** 1 Orthopedic Surgery/Neurological Surgery, Campbell University School of Osteopathic Medicine, Lillington, USA; 2 Neurological Surgery, Campbell University School of Osteopathic Medicine, Lillington, USA; 3 Neurosurgery/Duke Global Neurosurgery and Neurology, Duke University Medical Center, Durham, USA

**Keywords:** anatomical corridor, mis, t-lif, t-miss, lumbar spine surgery, kambin prism, kambin triangle

## Abstract

Kambin’s triangle has become the anatomical location of choice when accessing the lumbar spine to treat degenerative spinal disorders. Currently, lumbar interbody fusion is the most common procedure utilizing this space; however, with the advent of the Kambin’s prism definition, advanced imaging modalities, and robotic-assisted techniques, lumbar spine surgery has become increasingly precise and less invasive. These technological and procedural advances have drastically reduced the rate of complications, improved patient outcomes, and expanded the use of the Kambin’s triangle to treat different pathologies utilizing cutting-edge techniques. In this review, the authors present the current uses of the Kambin’s triangle and the future application of this anatomical corridor in lumbar spine surgery.

## Introduction and background

Lower back pain is a leading cause of disability in the United States, resulting in a substantial burden on both patient quality of life and healthcare costs [1]. As the United States’ population continues to age, the need for operative treatment of lumbar pathologies will grow exponentially. The proportion of the US population over the age of 65 is projected to increase from 12% in 2000 to 20% by 2030 [2]. This is associated with an increase in the prevalence of degenerative spinal disorders, leading to an increase in the need for surgical treatment of these conditions [2].

Multiple novel surgical techniques have been employed in recent years to adequately treat spine pathologies while mitigating perioperative morbidity associated with traditional spine surgery. Some of these techniques include minimally invasive spinal surgeries (MISS) and endoscopic procedures, percutaneous fixation, osteobiologic use, and expandable bone grafts. Among these, transforaminal MISS (t-MISS) through the Kambin’s triangle is the standard of care for lumbar degenerative diseases [3]. However, this technique requires great dexterity given the limited visualization of the surgical field and narrow operation window [4-6]. Consequently, a good understanding of Kambin’s triangle is paramount to the success of t-MISS.

**Figure 1:**
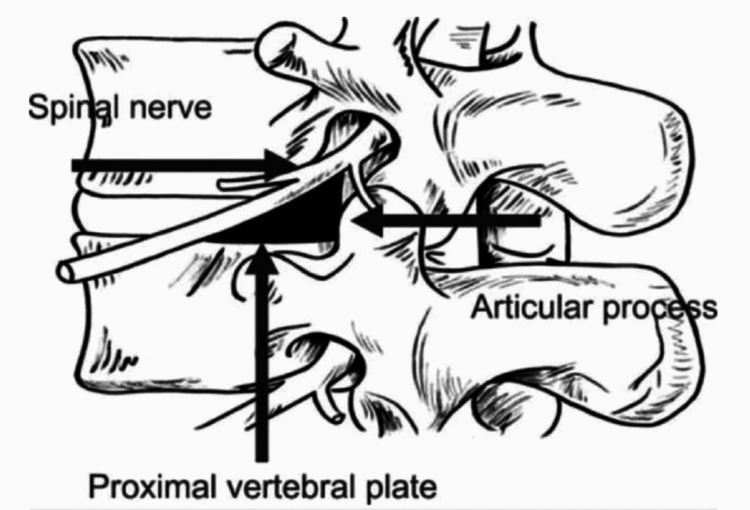
Anatomical boundaries of the Kambin's triangle Image adapted from Hoshide R, Feldman E, Taylor W. Cadaveric analysis of the Kambin's triangle. Cureus. 2016 Feb 2;8(2):e475. DOI: 10.7759/cureus.475. License CC BY 3.0.

In the current literature, several studies have investigated the anatomic details of Kambin's triangle [7-9], originally defined as a right triangular working zone of the lumbar intervertebral foramen bounded inferiorly by the proximal plate of the lower lumbar segment, anteriorly by the exiting nerve root, posteriorly by the proximal articular process of the inferior vertebra, and medially by the dura mater (Figure 1) [7,10-13]. Since a triangle is typically three-sided, the presence of four boundaries in the Kambin's triangle has led to the description of the Kambin prism [8]. Although it is well agreed that a thorough understanding of foraminal anatomy (including Kambin’s triangle) is important for the success of t-MISS, fewer studies have been successful at reconstructing a three-dimensional (3D) image of the Kambin’s triangle [12,14,15]. This 3D reconstruction is important for surgical planning and real-time navigation in the t-MISS operating field [16].

Here, we present the most up-to-date review on the 3D reconstruction of Kambin’s triangle, and a preview of the current and next era of spinal surgical management through the triangle.

## Review

Kambin’s triangle reconstruction

Few studies have attempted to accurately reconstruct the Kambin’s triangle through image segmentation using both computed tomography (CT) and magnetic resonance imaging (MRI) [17-19]. For instance, Huang et al. used a CT image segmentation technique to create a 3D model of the lumbar vertebra [20]. Unfortunately, many other segmentation studies aiming at recreating lumbar structures focused on bones [20-22], discs [23], and spinal muscles [19]. To the authors’ knowledge, only one study succeeded in recreating a comprehensive 3D model of the Kambin’s triangle including bones, dura mater, discs, and nerve roots [16]. In their study, Su et al. used a combination of 3D CT/MRI fusion imaging and a deep learning model based on 3D U-Net to effectively segment spinal structures simultaneously on MRI, thus generating an accurate 3D model of the Kambin’s triangle. Although this model has not been largely tested, it presents an important step for clinical analysis and surgical planning.

Anatomical variations and considerations

Although Kambin’s triangle has long been considered a safe anatomical corridor and has now become a common entry point for many procedures of the lumbar spine, it should be noted that studies have also shown variations that need to be considered. As the triangle can be located at different levels of the lumbar spine, there are multiple reports regarding the area of the triangle at different levels of the spine, concluding that from L1 to L5, the area of Kambin’s triangle increases, with the maximum area being at the L4-5 level [24-27]. This general pattern may aid surgeons in surgical planning. It has also been reported that there is significant variation between individuals when measuring the area [28]. A study by Ozer et al. demonstrated that only 6/34 studied patients had a classical Kambin’s triangle, while many had narrowed or even no space within the triangle [28]. In addition to variations in the size of the triangle, surgeons must also consider atypical anatomy, which may expose critical vasculature. A 2015 cadaveric evaluation of the safety of Kambin’s triangle demonstrated that, although described as a space relatively free of neuro-vasculature, it was not uncommon to find intervertebral and ascending lumbar vein branches within the triangle [29]. Arterial content, including the artery of Adamkiewicz, has also been reported in up to 20% of cases [30]; however, it should be noted that this artery is usually above L3 and is only found in 2% of cases when using the Kambin’s prism definition to assign a border to all relevant anatomical structures [8].

With literature reports of variations in the Kambin’s triangle and the possibility of vasculature in this area, the triangle still remains a common entry point for accessing the lumbar spine. Advanced imaging techniques and careful preoperative planning may be employed to account for these anatomic variations and ensure patient safety. Furthermore, angiographic imaging and consultation with a vascular surgeon may be necessary to confirm that the patient does not have important vasculature in the operating area.

Present uses

In current practice, Kambin’s triangle is used for a variety of procedures (Table 1), including endoscopic surgery through a corridor in the intact spine and assessing disc space after complete or partial facetectomies [8]. However, lumbar interbody fusion (LIF) is by far the most common procedure.

**Table 1:** Techniques utilizing the Kambin’s triangle LIF: lumbar interbody fusion; MIS: minimally invasive surgery; percTLIF: percutaneous transforaminal lumbar interbody fusion.

Techniques
Transforaminal minimally invasive spinal surgeries (t-MISS)
Robotic-assisted percTLIF
percTLIF with an expandable titanium cage
MIS extraforaminal LIF
Percutaneous vertebroplasty

Lumbar Fusion

Spinal fixation procedures have seen an incredible advancement in techniques and technologies in the past decades. Prior to the use of Kambin’s triangle for treating lumbar spine pathologies, postoperative bleeding, tissue disruption, and neurovascular complications were common in these procedures [31,32]. Following the introduction of Kambin’s triangle approach, lumbar fusion saw a shift from a posterolateral approach to the current transforaminal interbody approach, which is now being used in combination with minimally invasive techniques.

Minimally invasive surgery (MIS) transforaminal lumbar interbody fusion (TLIF) is the standard of care when treating lumbar spine pathologies using Kambin’s triangle [33]. There is a dearth of evidence suggesting the superiority of MIS-TLIF to traditional open TLIF. As such, MIS-TLIF has been associated with decreased pain, blood loss, and hospital length of stay, compared to open TLIF, leading to decreased healthcare costs [34-36].

Future applications

As it is becoming clear that the future of spinal surgery will focus on value-driven care and minimally invasive techniques to reduce costs and improve patient outcomes, novel techniques for improving these surgeries are increasing. With the acceptance of Kambin’s triangle as a safe entry point for access to elements of the spine, newer methods for improving techniques are being reported such as mapping variations in Kambin’s triangle preoperatively, creating techniques that reduce tissue disruption, and using advanced technology to improve surgical outcomes.

Preoperative Evaluation of Kambin’s Triangle

As previously discussed, multiple studies have shown that there are anatomic variations in the dimensions of Kambin’s triangle based on the spinal level, which affects the angle of entry [25,29]. Consequently, there has been an interest in improving and evaluating preoperative imaging techniques to improve patient outcomes. Description of the use of 3D CT/magnetic resonance fusion imaging technique to evaluate Kambin’s triangle found that the use of this technique preoperatively to acquire accurate measurements can enhance patient safety and outcomes in transforaminal endoscopic lumbar discectomy [37]. A more recent study used the same method with artificial intelligence (AI) to reduce processing time and evaluation of these preoperative studies, showing the importance of incorporating AI and deep learning to plan personalized and technically precise surgeries in the future [15].

Expanded Intraoperative Techniques

Although preoperative advances in the evaluation of the Kambin’s triangle are important, much of the recent literature has focused on future directions in expanding its use. These potential uses include treatments of pathologies such as upper lumbar fracture with neurologic deficit, osteoporotic vertebral compression fractures, and thoracolumbar burst fractures [38-41]. In these instances, the Kambin's triangle has been used to guide safe bone decompression, needle and wire placement, and endoscopic cannula docking. These studies are important as they employ this anatomical safe triangle in different applications, which could reduce the overall cost of care and improve patient outcomes, as it has done for common procedures such as LIF.

Future Improvements to LIF via Kambin’s Triangle

The bulk of recent literature involved in potential future uses of Kambin’s triangle remains focused on improving LIF. As these procedures become less invasive, there is an increasing dependence on the use of the endoscope as the primary tool to facilitate these procedures. A recent paper described the use of fully endoscopic lumbar surgery to treat spinal canal stenosis [42]. The full-endoscopic technique was able to not only perform LIF using an interbody cage but to use the endoscope to achieve decompression via facetectomy, with excellent (80%) and good (20%) outcomes in patients [42]. Shortly after, Wang et al. published a case series discussing the use of percutaneous lumbar interbody fusion (percLIF) with an expandable titanium cage, the first to demonstrate the use of the percutaneous approach, which, unlike minimally invasive techniques, does not require an endoscope or facetectomy [43]. This study reported that percLIF could be used to treat grade 1 lumbar spondylolisthesis, reducing blood loss (when compared to MIS) and thus reducing the length of hospital stay (thereby reducing cost). Previous studies found high rates of postoperative complications when using traditional techniques to treat lumbar pathologies; however, with the use of free-running electromyography (EMG), the authors demonstrated 90% fusion one-year postoperative with minimal complications [43]. It has also been previously shown that the percLIF approach can even be used for the same procedure under awake anesthesia, reducing costs and improving patient outcomes further [44]. As less invasive techniques such as percLIF emerge, surgeons must become more precise in planning and executing surgical procedures given the challenging anatomical variation in Kambin’s triangle. This could benefit from robot-assisted (RA) techniques, which have achieved high precision in pedicle screw placement [45]. Literature on the use of RA in accessing the Kambin’s triangle is rare; to our knowledge, Dalton et al. reported the first case series describing RA trajectory into Kambin’s triangle [45]. This study resulted in no complications and limited length of hospital stay, suggesting a place for robot-guided techniques in LIF using Kambin’s triangle during percLIF [45].

## Conclusions

There is no doubt that the anatomic definition of Kambin’s triangle was a turning point in the management of spinal pathologies. This anatomical safe triangle is associated with lower complications and reduced operating time. Furthermore, it promoted technological advancements that have made minimally invasive techniques feasible and common today. As minimally invasive procedures have been proven to not only improve patient care but also reduce the cost of health care for patients, there has been a focus on expanding the use of and improving techniques that utilize the Kambin’s triangle. With the more recent definition of Kambin's prism, there is now a push to pioneer safer and less invasive techniques when treating lumbar pathology. Currently, Kambin's triangle is most commonly used in lumbar interbody fusion techniques though there are various procedures in which it can be applied. The introduction of Kambin’s triangle approaches created a shift from posterolateral techniques to the current transforaminal interbody approach used in conjunction with minimally invasive techniques. MIS-TLIF is the standard of care when using Kambin’s triangle and there is empirical evidence advocating for its superiority against traditional open TLIF.

Following well-established techniques for minimally invasive procedures via Kambin’s triangle, recent studies have begun to focus on a percutaneous approach to treating lumbar spine pathologies via the Kambin’s triangle. This approach further minimizes tissue disruption and presents a good example of the constant innovation surrounding the use of Kambin’s triangle. In addition to the percutaneous technique, Kambin's triangle is being used in pilot studies to treat new pathologies with excellent outcomes. As these new applications are studied further, innovative techniques will be aided as the use of advanced imaging and robotics is further implemented in procedures. Thus, we conclude that the use of the Kambin’s triangle in lumbar pathologies will continue to have expanded indications and prior applications will become more efficient as the use of more advanced technologies (such as RA techniques) become the mainstay of these procedures.
